# Demand for Intensive Care beds and patient classification according to the priority criterion*

**DOI:** 10.1590/1518-8345.4945.3489

**Published:** 2021-10-29

**Authors:** Aline Nassiff, Mayra Gonçalves Menegueti, Thamiris Ricci de Araújo, Maria Auxiliadora-Martins, Ana Maria Laus

**Affiliations:** 1 Universidade de São Paulo, Escola de Enfermagem de Ribeirão Preto, PAHO/WHO Collaborating Centre for Nursing Research Development, Ribeirão Preto, SP, Brazil.; 2 Universidade de São Paulo, Faculdade de Medicina de Ribeirão Preto, Ribeirão Preto, SP, Brazil.

**Keywords:** Triage, Nursing, Intensive Care Units, Health Management, Patients, Health Services Needs and Demand, Triaje, Enfermería, Unidades de Cuidados Intensivos, Gestión em Salud, Pacientes, Necesidades y Demandas de Servicios de Salud, Triagem, Enfermagem, Unidades de Terapia Intensiva, Gestão em Saúde, Pacientes, Necessidades e Demandas de Serviços de Saúde

## Abstract

**Objective:**

to assess the demand for Intensive Care Unit beds as well as the classification of the patients for admission, according to the priority system.

**Method:**

a retrospective and cross-sectional study, developed from January2014 to December2018 in two Intensive Care Units for adults of a university hospital. The sample consisted of the requests for vacancies according to the priority system(scale from 1 to 4, where 1 is the highest priority and 4 is no priority), registered in the institution’s electronic system.

**Results:**

a total of 8,483 vacancies were requested, of which 4,389(51.7%) were from unitB. The highest percentage in unitA was of Priority2 patients(32.6%); and Priority1 was prevalent in unitB(45.4%). The median lead time between request and admission to unitA presented a lower value for priority1 patients(2h57) and a higher value for priority4 patients(11h24); in unitB, priority4 patients presented shorter time(5h54) and priority3 had longer time(11h54). 40.5% of the requests made to unitA and 48.5% of those made to unitB were fulfilled, with 50.7% and 48.5% of these patients being discharged from the units, respectively.

**Conclusion:**

it is concluded that the demand for intensive care beds was greater than their availability. Most of the patients assisted were priorities1 and2, although a considerable percentage of those classified as priorities3 and4 is observed.

## Abstract (es)

**Objetivo:**

evaluar la demanda de camas de la Unidad de Terapia Intensiva, así como también la clasificación de los pacientes para su ingreso, según el sistema de prioridades.

**Método:**

estudio retrospectivo, transversal, realizado en dos Unidades de Terapia Intensiva de adultos de un hospital universitario, de enero de 2014 a diciembre de 2018. La muestra estuvo conformada por solicitudes de vacantes según el sistema de prioridades (escala de 1 a 4, donde 1 es alta prioridad y 4 sin prioridad), registrados en el sistema electrónico de la institución.

**Resultados:**

se solicitaron 8.483 plazas, de las cuales 4.389 (51,7%) pertenecían a la unidad B. El mayor porcentaje en la unidad A era de pacientes de Prioridad 2 (32,6%); y de Prioridad 1 en la unidad B (45,4%). La mediana del tiempo de espera entre la solicitud y el ingreso a la unidad A fue menor para los pacientes con prioridad 1 (2h57) y mayor para los pacientes de prioridad 4 (11h24); en la unidad B, los pacientes de prioridad 4 tuvieron un tiempo más corto (5h54) y los de prioridad 3 uno más largo (11h54). El 40,5% de las solicitudes realizadas a la unidad A y el 48,5% a la unidad B fueron autorizadas, mientras que el 50,7% y el 48,5% de estos pacientes, respectivamente, fueron dados de alta de las unidades.

**Conclusión:**

se concluye que la demanda de camas de terapia intensiva fue mayor que su disponibilidad. La mayoría de los pacientes atendidos fueron de prioridad 1 y 2, aunque se observó que un porcentaje considerable de ellos fue clasificado como de prioridad 3 y 4.

## Abstract (pt)

**Objetivo:**

avaliar a demanda por leitos de Unidade de Terapia Intensiva bem como a classificação dos pacientes para admissão, segundo o sistema de prioridades.

**Método:** estudo retrospectivo, transversal, desenvolvido em duas Unidades de Terapia Intensiva adulto de hospital universitário, de janeiro de 2014 a dezembro de 2018. A amostra constituiu-se das solicitações de vagas segundo o sistema de prioridades (escala de 1 a 4, sendo 1 maior prioridade e 4 nenhuma prioridade), registradas no sistema eletrônico da instituição.

**Resultados:**

foram solicitadas 8.483 vagas, das quais 4.389 (51,7%) eram da unidade B. O maior percentual na unidade A foi de pacientes Prioridade 2 (32,6%); e de Prioridade 1 na unidade B (45,4%). O tempo mediano de espera entre a solicitação e admissão na unidade A apresentou menor valor para pacientes da prioridade 1 (2h57) e maior valor naqueles de prioridade 4 (11h24); na unidade B, pacientes de prioridade 4 apresentaram menor tempo (5h54) e maior na prioridade 3 (11h54). Foram atendidas 40,5% das solicitações feitas à unidade A e 48,5% para a unidade B, sendo que, 50,7% e 48,5% destes pacientes, respectivamente, receberam alta das unidades.

**Conclusão:**

conclui-se que a demanda por leitos de terapia intensiva foi maior que sua disponibilidade. A maioria dos pacientes atendidos foram prioridades 1 e 2, embora se observe um percentual considerável daqueles classificados em prioridades 3 e 4.

## Introduction

Over the years, a global trend has been identified in the increasing need for intensive care beds, resulting from population growth and aging and increased long-term survival of patients with chronic diseases associated with episodes of acute diseases, as well as in the change in the perception of the profile of the patients who would benefit from admission to critical care services, producing a sustained and growing demand for these services^(1-2)^.

The expansion of the Intensive Care Units(ICU) due to the pressure to expand the number of beds is complex and relatively limited, influenced by a set of factors that involve a greater supply of resources, which are costly, and which exceed the bed dimension, for requiring considerable investment in human resources, specialized equipment and supplies^(3)^.

This mismatch between demand and supply of ICU beds imposes the need for resource screening and allocation. Frequently, high-risk decisions need to be made in a setting where there is significant documented variability on how these beds are managed. Limited patient information, uncertainty about possible outcomes, and extreme pressure for quick decision-making exacerbate this process for the professionals^(4-6)^.

Worldwide, several classification and screening systems have been developed and tested with the purpose of supporting the criteria for admission to an ICU. However, there are no conclusive studies that show consensus on comprehensive and definitive courses of action for admission to the sector, which limits the strength of the recommendations. The screening processes are characterized by cultural and regional variability and are influenced by the institutional context. Consequently, in this scenario, the working time, clinical experience and judgment of the intensivist need to be considered as impact variables in the decision-making process^(7)^.

Admission to the ICU can be based on different models. In the prioritization model, the patients are categorized into priority levels based on their likelihood of benefiting from admission to this unit, which varies from major benefit to no benefit. In the diagnosis-based model, the analysis of specific conditions or diseases will determine the need for admission. On the other hand, the model based on objective parameters includes laboratory tests and vital signs, dysfunctions found in imaging and electrocardiogram tests, as well as clinical assessment to determine the need for admission^(8)^.

In the practice, the availability and need for ICU beds changes dynamically, according to the impact of time variability, which affects the capacity of this unit in terms of its functioning. In this scenario, the work by the managers of these units, a space predominantly occupied by nurses, is carried out under an atmosphere of permanent challenge, given the need to plan the necessary resources and equip the unit so that assistance and care provision can occur safely and with high clinical quality.

The adverse consequences of this exhaustion of the ICU ability to meet multiple demands can be mitigated through a better understanding of this process, which needs to be grounded on clear criteria that assist in patient admission decisions.

The literature review indicated few studies that recommend the prioritization system as a criterion for admission to intensive care units, as well as those that provide representative data on the demand for beds in these units. Given the worldwide phenomenon of growing demand for intensive care, this study aimed at assessing the demand for Intensive Care Unit beds, as well as the classification of the patients for admission, according to the priority system.

## Method

### Study design

This is a retrospective and cross-sectional study.

### Locus

It was developed in a large-size public teaching hospital, a reference in regional health care in the State of São Paulo, located in Ribeirão Preto, Brazil, with 900 beds. The institution has specialized units with high-complexity beds distributed in post-operative cardiac and neurological surgery ICU(10 beds), cardiology ICU(22 beds), clinical and general surgery ICU A(09 beds); and an ICU B for patients in urgent and emergency situations(16 beds).

The study scope was to analyze the ICU in which the bed request prioritization system is implemented. In this way, two Intensive Care Units were selected, herein named ICUA and ICUB. ICUA, intended for assistance to adult patients, in various medical specialties, in clinical or surgical hospitalization, underwent an expansion in October2018, rising from 09 to 13 beds. ICUB is devoted to the care of patients in urgent and emergency situations, particularly trauma victims.

### Period

The data collected refer to the period from January1^st^,2014 to December31^st^,2018.

### Selection criteria

The study object consisted of the requests for inpatient vacancies in the intensive care units, for patients aged 18 years or over, regardless of gender, type of treatment, diagnosis, or unit/specialty, registered in the institution’s electronic system.

### Study variables

The requests are made in the system by the specialty physician, by filling out a form with information regarding the patient’s data; previous diseases, through the International Classification of Diseases(ICD-10), reason for admission, objective parameters(mechanical ventilation, use of vasoactive drugs and altered state of consciousness), observations and care unit(ICUA or ICUB).

Such information is analyzed and evaluated by an intensive care physician, who classifies the request according to Priority(P) criteria on a scale from one to four. This scale was adapted by the institution from the model of five Priorities of Ministerial Ordinance No.895, dated March31^st^,2017^(9)^. According to the unit’s criteria, the definition of each priority is as follows: Priority1-Patients who need life support interventions, with a high probability of recovery and without any therapeutic support limitation; Priority2-Patients who need intensive monitoring, due to the high risk of needing immediate intervention, and without any therapeutic support limitation; Priority3-Patients who need intensive monitoring, due to the high risk of needing immediate intervention, but with limited therapeutic intervention; Priority4-Patients with a terminal disease or dying, with no possibility of recovery. In general, these patients are not indicated for admission to the ICU(unless they are potential organ donors). However, their admission can be exceptionally justified, considering the peculiarities of the case and subjected to the intensive care physician’s discretion.

The analysis period should not exceed 48 hours and, if not analyzed, it was automatically canceled by the system, generating the need for a new request. All the information related to the vacancy request process is stored in the institution’s database, which was made available by the Information and Analysis Center.

A high number of concomitant requests was verified, for ICUA and ICUB, for the same patient, but with different priority classification and, in these cases, it was decided to consider the one whose priority indicated greater probability of recovery.

### Data treatment and analysis

The data obtained by the Information and Analysis Center were entered by the main researcher into Excel spreadsheets where they were coded and categorized for statistical analysis. The data collected were entered into Microsoft Excel365^®^ version2019 spreadsheets and analyzed according to descriptive statistics using the SPSS^®^(*Statistical Package for the Social Sciences*) program, version24 for Windows. Descriptive simple frequency analyses were used for nominal or categorical variables and mean or median, depending on data distribution, for continuous variables. The data related to the bed occupancy rates during the study period were organized using a historical series.

### Ethical aspects

The study was submitted and approved by the Research Ethics Committees of the Ribeirão Preto Nursing School/USP and of the institution studied, under CAAE protocol83189718.4.3001.5440, and was developed so as to ensure compliance with the precepts set forth in Resolution466/2012 of the National Research Ethics Committee.

## Results

From a total of 10,028 vacancy requests for ICUA or ICUB in the period studied, 1,545(15.4%) were incomplete, with regard to the criterion for indication; therefore, 8,483(84.6%) requests were analyzed. There was predominance of male patients(54.3% in ICUA and 62.3% in ICUB). The predominant age group was 60-79 years old for ICUA(44%) and ICUB(40.1%), followed by 46-59 years old with 25% and 24.6%, respectively.

The mean annual bed occupancy rates during the period for ICUA were 86.2%, 91%, 89.8%, 91.2% and 96.7%; and, for ICUB, 98.4%, 97.2%, 97.7%, 98.2% and 96.7%. As for the number of requests for vacancies in the period under study, in ICUA they were 836, 889, 886, 857 and 626 and, in ICUB, they were 1,041, 986, 745, 835 and 782. There was a reduction in the number of requests over the years, more pronounced in2018. In ICUA, this reduction was 27% compared to the previous year.

The number and distribution of the requests according to the prioritization established by the intensivist are presented in Table 1.

**Table 1:** Distribution of the bed requests by institution, according to the priority criterion. Ribeirão Preto, São Paulo, Brazil,2014-2018

Priority	ICU^†^ 1		ICU^†^ 2		TOTAL	
	f*	%	f*	%	f*	%
Priority 1	791	19.3	1,994	45.4	2,785	32.8
Priority 2	1,319	32.2	1,431	32.6	2,750	32.4
Priority 3	1,009	24.6	467	10.6	1,476	17.4
Priority 4	555	13.6	343	7.8	898	10.6
Not informed	420	10.3	154	3.5	574	6.8

* Frequency;

† Intensive Care Unit

In general, it was verified that ICUB presented a higher number of requests in priority1(45.4%), when compared to ICUA(19.3%). This difference can be explained by the characteristics of the unit, since ICUB concentrates urgency and emergency care, receiving patients who are, in their vast majority, young people and trauma victims. From this perspective, it enables greater benefit potential from intensive care. As for ICUA, the profile of the patients referred is related to those for the treatment of comorbidities, which explains the high number of requests classified as P3 and P4, which together accounted for38.2%.

The calculated waiting times(in hours) for an intensive care bed, from the request, are described in Table 2.

**Table 2:** Waiting time for a bed in ICUA and ICUB, according to the priority criterion. Ribeirão Preto, São Paulo, Brazil, 2014-2018

Intensive Care Unit A				Intensive Care Unit B			
Priority	Median	Minimum	Maximum	Priority	Median	Minimum	Maximum
Priority 1	2h57	0	59h24	Priority 1	7h18	0	92h48
Priority 2	5h51	0	96h24	Priority 2	8h48	0	93h42
Priority 3	8h36	0	152h24	Priority 3	11h54	1h.	57h12
Priority 4	11h24	1h09	61h24	Priority 4	5h54	0	62h30
Not informed	6h30	1h30	20h06	Not informed	27h36	27h36	27h36

In ICUA, the lowest median value for the waiting time is related to the classification of priority1(2h57min) and the highest value is related to priority4(11h24min). For ICUB, the lowest median time value was 5h54min for priority4 care, followed by 7h18min(priority1) and by 8h48min(priority2).

In relation to the fulfillment status of the requests, it is verified that, both in ICUA and in ICUB, the percentage was less than 50%, as described in Table 3.

**Table 3:** Distribution of the bed requests in ICUA and ICUB, according to priority and situation of care. Ribeirão Preto, São Paulo, Brazil,2014-2018

	Intensive Care Unit A							Intensive Care Unit B					
	Met		Not met		Total			Met		Not met		Total	
Priority	f*	%	f*	%	f*	%	Priority	f*	%	f*	%	f*	%
Priority 1	603	76.2	188	23.8	791	100	Priority 1	1,545	77.5	449	22.5	1,994	100
Priority 2	717	54.4	602	45.6	1,319	100	Priority 2	514	35.9	917	64.1	1,431	100
Priority 3	295	29.2	714	70.8	1,009	100	Priority 3	50	10.7	417	89.3	467	100
Priority 4	38	6.8	517	93.2	555	100	Priority 4	21	6.1	322	93.9	343	100
Not informed	5	1.2	415	98.8	420	100	Not informed	1	0.6	153	99.4	154	100
Total	1,658	40.5	2,436	59.5	4,094	100	Total	2,131	48.6	2,258	51.4	4,389	100

* Frequency

It is verified that both units were able to partially meet the requests, with the greatest number being those categorized as having the highest clinical priority. They presented differentiation in the reception of priority2, whose profile of patients should be those who are in an acute condition, but with a good clinical prognosis.

Given this situation, the outcome presented by the patients was verified, separated according to the classification established by the intensive care physician, and presented in Table 4.

**Table 4:** Distribution of the bed requests in ICUA and ICUB, according to priority and to hospitalization outcome. Ribeirão Preto, São Paulo, Brazil,2014-2018

	Intensive Care Unit A							Intensive Care Unit B					
	DISCHARGE		DEATH		TOTAL			DISCHARGE		DEATH		TOTAL	
Priority	f*	%	f*	%	f	%	Priority	f*	%	f*	%	f*	%
Priority 1	577	72.9	214	27.1	791	100	Priority 1	1,308	65.6	686	34.4	1,994	100
Priority 2	724	54.9	595	45.1	1,319	100	Priority 2	683	47.7	748	52.3	1,431	100
Priority 3	385	38.2	624	61.8	1,009	100	Priority 3	187	40.0	280	60.0	467	100
Priority 4	387	69.7	168	30.3	555	100	Priority 4	193	56.3	150	43.7	343	100
Not informed	168	40.0	252	60.0	420	100	Not informed	67	43.5	87	56.5	154	100
Total	2,241	54.7	1,853	45.3	4,094	100	Total	2,438	55.5	1,951	44.5	4,389	100

* Frequency

The percentages of deaths among the patients prioritized as1 and2 occurred in greater numbers in ICUB when compared to ICUA. In Table 5, it is possible to see these outcomes in relation to the situations of inpatient care.

**Table 5:** Distribution of the bed requests in Intensive Care UnitsA andB, according to the situation of care and to the hospitalization outcome. Ribeirão Preto, São Paulo, Brazil,2014-2018

	Intensive Care Unit A							Intensive Care Unit B					
	DISCHARGE		DEATH		TOTAL			DISCHARGE		DEATH		TOTAL	
Situation	f*	%	f*	%	f*	%	Situation	f*	%	f*	%	f*	%
Met	840	50.7	818	49.3	1,658	100	Met	1,290	60.5	841	39.5	2,131	100
Not met	1,401	57.5	1,035	45.1	2,436	100	Not met	1,148	50.8	1,110	49.2	2,258	100
Total	2,241	54.7	1,853	45.3	4,094	100	Total	2,438	55.5	1,951	44.5	4,389	100

* Frequency

It is possible to identify that, among the requests for admission to the intensive care unit that were met, patient discharge occurred in higher percentages in ICUB, which presented better rates when compared to ICUA. However, it was verified that, in the required and not met hospitalizations, in both units, the patients evolved to discharge from the institution, with values above50%.

In the group of patients evaluated during the research period, the reasons for hospitalization were similar in both units, with the most frequent ones being “lung or airway disease”, “neurological disease”, “cardiovascular disease” and “postoperative”.

## Discussion

This research sought to assess the panorama of requesting vacancies for the intensive care unit and the service capacity given the availability of beds.

Considering the illness profile of the population today, access to hospitalization in intensive care beds is vital, since it has been shown that critically-ill patients need early interventions to improve their clinical outcomes. When the number of patients requiring intensive care is greater than the number of beds available, there is a limitation in terms of positive therapeutic opportunity.

In this study, non-admission to intensive care units due to unavailability of beds was common, as the requests were met in 48.5% in ICUA and in 48.6% in ICUB. The mean operational occupancy rate is higher than that recommended by the Ministry of Health, which proposes to maintain it between 80%and85%^(10)^. A review of the international literature that explored the operationalization and assessment of bed occupancy in intensive care pointed out a significant variation in the calculations and, although there is no definitive evidence, values around 70%to75% in occupancy levels were identified^(11)^.

As for gender, there was predominance of male patients. This result reflects a higher rate of illness due to serious and chronic conditions in men, which can be associated with the concept that taking care of their own health is not a practice valued by males, linked to cultural and social aspects^(12)^. Health risk behaviors among men can be attributed to the lower use of health services, mainly those of a preventive nature, associated with the values around which they build their masculinity, factors that can determine the forms of illness and death^(13)^. Thus, for being part of an emergency unit, ICUB receives an even higher percentage of male patients, particularly trauma victims.

Over the period investigated, a drop was verified in the number of requests for admission to both study units, particularly in2018. The implementation of palliative care services in the institution may have contributed to the reduction in the number of requests for ICU beds for those patients with a poor prognosis and an indication for exclusive palliative care. Another aspect refers to the expansion of beds in the Coronary Unit and Postoperative Intensive Care Unit, influencing these numbers of requests.

In this study, it was identified that in ICUA, less than 20% of the requests corresponded to priority1, and the sum of this with those of priority2 accounted for 51.4% of the total. In the case of ICUB, this percentage was more significant, as it totaled78%. Such a scenario may suggest the need for investments in more assertive guidance by the medical team of the inpatient units regarding the need for a critical unit for patient care. Furthermore, it should be considered that the admission of patients classified as priority4 is not expected, as they do not have recovery and survival chances.

In the context of a vacant bed, the admission of these patients who have a complication due to the underlying disease can directly impact on the admission of patients with a better prognosis, who could benefit from intensive care. However, there is a need to assess the exceptionality of situations experienced in the daily routine of the units, such as a patient in palliative care due to a disease without the possibility of curative treatment. Having the disease under control, when presenting a new acute disease, and having the possibility of recovery, would characterize prioritization for admission to intensive care. A number of researchers point out that some intensive palliative treatments can be better conducted in an ICU when the patients are in crisis, with the possibility of stabilizing an acute condition, justifying their hospitalization in this unit^(14)^.

The unavailability of beds in the ICU determines the permanence of critically-ill patients in wards, which may lead to complications arising from critical conditions and/or new sequelae of their chronic diseases, as well as the risk of late recognition of the deterioration of their clinical condition. Critically-ill patients need early interventions to improve their clinical condition^(15-16)^.

Early admission to the ICU has been identified as a standard of successful intensive care practices, as it provides the opportunity for the patient to be admitted in a better clinical condition and, consequently, to have a better outcome. Conversely, delays in transfers to the ICU can lead to increased length of stay and mortality^(17)^. In this study, we identified that in ICUA, the median waiting time to meet the request for a vacancy was 2.95 hours for priority1 and 7.3 hours for ICUB. The high number of hours for patients classified as Priority1 to be admitted to the ICU can compromise their evolution. A study developed in2016^(18)^ identified a 3% increase in the chances of in-hospital death, associated with each hour of waiting time for an ICU bed in the ward. Another research study that evaluated the impact of late ICU admission on mortality, given the immediate unavailability of a bed, found a 1.5% increase in mortality per hour of waiting for admission to the ICU^(15)^.

The limitation of hospitalization in intensive care invariably impacts on the permanence of critically-ill patients being cared for in wards, which do not always have specialized human and technological resources, in addition to modifying the patients’ susceptibility to adverse events^(3)^. Providing critical care outside the scope of intensive care implies an increase in the workload and stress level on the part of workers, especially in the Nursing area. It must also be considered that critical treatments conducted within the scope of regular hospitalizations by the SUS cause high additional costs that end up being absorbed by the institution, burdening its financial resources^(15)^.

A research study^(19)^ with the purpose of analyzing the epidemiological and clinical characteristics of critically-ill patients who were denied admission to the ICU due to the unavailability of beds and estimating the direct costs of treatment during this period, identified prognostic scores, such as high values in *Acute Physiology and Chronic Health Evaluation* II(APACHEII), *Sequential Organ Failure Assessment* (SOFA) and Therapeutic Intervention Scoring System *(*TISS), showing a high degree of organ dysfunction with the need for interventions that included the use of mechanical ventilation and vasoactive drugs, associated with high costs and unfavorable prognosis.

In relation to the patients coming from the emergency units and requiring rapid admission to the ICU, waiting for admission to this unit can result in longer hospitalization time and ventilatory support. For patients with acute respiratory failure, a waiting time for admission to the ICU of more than one hour was considered a predictor of mortality^(20)^.

A study^(21)^ that aimed at evaluating the influence of the time interval between admissions to the emergency unit and the ICU on mortality concluded that the waiting time for admission to the ICU is associated with the death outcome.

The results of this study showed that, in line with the prioritization protocol, there was low fulfillment of the requests for patients classified as priority4. However, it can be identified that in ICUB, when these requests were met, the median waiting time was shorter than that of priority1 patients.

The data collected alone cannot capture the breadth of circumstances in which priority4 patient admissions occurred. However, those patients with diseases without a cure prognosis can present some acute impairment and benefit from ICU care. This can be seen in the discharge percentage presented for these patients(56.3%).

The decisions about when to admit a patient to the ICU are extremely challenging, multifaceted, involving a dynamic and complex process, and must be made in a stressful and emotionally charged environment, in a short period of time. The severity, reversibility potential of the acute disease, presence and severity of comorbidities, the patient’s age and bed availability are factors that influence the decision-making process^(4)^.

In ICUA, the discharge percentage in priority4 was 69.7%, even with a median waiting time of 11.4 hours, higher than all other prioritizations. Likewise, the justification for hospitalization could be attributed to the presence of an acute disease, consequently with the possibility of a cure, or even due to a discrepancy in the prioritization performed by the intensivist.

It was observed that the vast majority of the patients classified as priority2 did not have their requests met. Considering that the priority categorization criteria give this group of patients a positive perspective in relation to the prognosis, non-fulfillment of the admission to the critical unit can lead to an unfavorable outcome.

The ideal ratio of ICU beds/population is that capable of ensuring that patients with the potential to benefit from admission to the ICU can be admitted. However, although the opening of new ICU beds seems the simplest solution, it may only be resolute in the short term^(1-3,22-23)^.

In order to determine the number of ICU beds needed by adult patients, so as to reduce waiting lines, a number of researchers evaluated 33,101 admission requests for 268 ICU beds, located in the state of Rio de Janeiro, over a one-year period. The results revealed that 25% of the patients were assisted, 55% abandoned the queue and 20% died, leading the authors to conclude that 628 intensive care beds would be needed to meet this demand and ensure that the maximum waiting time was 6 hours^(24)^.

A retrospective study^(25)^ carried out with the objective of drawing a relationship between mortality and the clinical and epidemiological profile of the patients admitted to the ICU evidenced a rate of 24.4%, with the highest percentage(53.3%) being obtained among the patients classified as priorityII by Society of Critical Care Medicine.

Intensive Care Units are considered key components in the care of patients at imminent risk of death and with a chance of recovery. The earlier the interventions in critically-ill patients are initiated, the better results can be obtained. However, the delay in bed availability in intensive care can be considered an important predictor of the patient’s clinical outcome.

The main limitation of this study was the use of a secondary data source, as gaps and inconsistencies were identified in the filling in of information in the application for vacancies in the electronic system, which resulted in the exclusion of 1,545(15%)requests.

The results of this study made it possible to identify problems related to the care of patients classified as priorities1 and2. Thus, patients affected by serious pathologies and with therapeutic possibility were partially contemplated for admission to the ICU.

## Conclusion

It is concluded that the demand for intensive care beds is greater than their availability; in addition to that, most of the patients assisted are priorities1 and2, although there is a considerable percentage of patients treated in priorities3 and4.
